# Laser Scissors and Tweezers to Study Chromosomes: A Review

**DOI:** 10.3389/fbioe.2020.00721

**Published:** 2020-07-16

**Authors:** Michael W. Berns

**Affiliations:** ^1^Beckman Laser Institute and Medical Clinic, University of California, Irvine, Irvine, CA, United States; ^2^Department of Biomedical Engineering, University of California, Irvine, Irvine, CA, United States; ^3^Department of Developmental and Cell Biology, School of Biological Sciences, University of California, Irvine, Irvine, CA, United States; ^4^Department of Surgery, School of Medicine, University of California, Irvine, Irvine, CA, United States; ^5^Institute of Engineering in Medicine, University of California, San Diego, San Diego, CA, United States; ^6^Department of Bioengineering, University of California, San Diego, San Diego, CA, United States

**Keywords:** laser, tweezers, scissors, chromosomes, mitosis, trapping

## Abstract

Starting in 1969 laser scissors have been used to study and manipulate chromosomes in mitotic animal cells. Key studies demonstrated that using the “hot spot” in the center of a focused Gaussian laser beam it was possible to delete the ribosomal genes (secondary constriction), and this deficiency was maintained in clonal daughter cells. It wasn’t until 2020 that it was demonstrated that cells with focal-point damaged chromosomes could replicate due to the cell’s DNA damage repair molecular machinery. A series of studies leading up to this conclusion involved using cells expressing different GFP DNA damage recognition and repair molecules. With the advent of optical tweezers in 1987, laser tweezers have been used to study the behavior and forces on chromosomes in mitotic and meiotic cells. The combination of laser scissors and tweezers were employed since 1991 to study various aspects of chromosome behavior during cell division. These studies involved holding chromosomes in an optical while gradually reducing the laser power until the chromosome recovered their movement toward the cell pole. It was determined in collaborative studies with Prof. Arthur Forer from York University, Toronto, Canada, cells from diverse group vertebrate and invertebrates, that forces necessary to move chromosomes to cell poles during cell division were between 2 and 17pN, orders of magnitude below the 700 pN generally found in the literature.

## Historical Background

Though the field of laser micro-irradiation to study cells structure and function is extensive, this review will focus on just one area: studies on chromosome structure and function.

Anecdotally, it is, perhaps interesting on how I entered this field when a graduate student in the turbulent 1960’s. Professor of Genetics Adrian Srb at Cornell University indicated the genetics department had just purchased a laser microscope and he thought it might be helpful in my research project. The system was the first commercial laser microscope ever built, and was made by Hadron Inc., Westbury, Long Island, N.Y. It utilized a pulsed ruby laser coupled to a microscope (see Figure 2.8 in Berns, 1974). The system was first applied to ablate a small region in the post-embryonic millipede that was suspected of being the region (anlagen) responsible for regulating the increase in body segments and legs as the immature millipede went through a series of molts to attain adulthood. Unfortunately, the laser ablation did not work, primarily because the red wavelength was so efficiently absorbed by the animal’s pigmented exoskeleton that the laser destroyed the animal. This avenue of approach was abandoned and instead, X-ray ablation of the anlagen was used (Berns and Keeton, 1968).

Though the initial experiments with the Hadron ruby laser microscope were a failure, I did become familiar with the scant literature on the use and the effects of laser radiation on biological systems. Invented in 1960 by Theodore Maiman at Hughes Aircraft (see Figure 3–9; Berns, 1974) and first coupled to a microscope by Marcel Bessis (Paris, France), the new field of laser microbeam irradiation was born. The first laser microscope was described by Bessis et al. (1962). He and his colleagues in Paris published numerous studies using the ruby laser microscope to alter and study blood cells as well as internal organelles such as mitochondria. However, due to the high energy output of the ruby laser, and a red wavelength that was naturally absorbed by certain cell organelles, studies were limited to short term experiments. Notwithstanding, it was the work of Bessis and others that motivated me and others pursue the use of a focused diffraction limited laser beam in cell and developmental biology.

## Scissors I: Genetic Surgery

**FIGURE 1:**
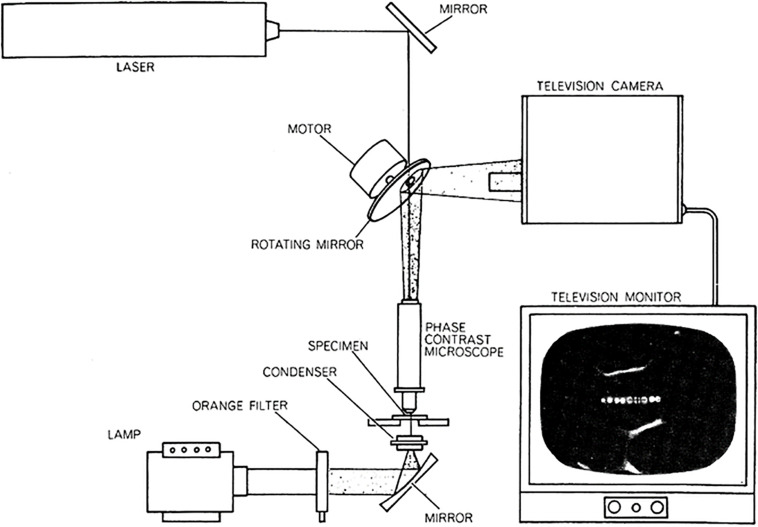
First argon ion laser microbeam microscope. The system utilized a Hughes Aircraft micro second pulsed blue-green (488 and 514 nm) argon ion laser. The 60 Hz pulsed beam passed through a 60 Hz rotating front-surface mirror into the microscope. The microscope image is reflected off the front surfaced mirror into an analog television camera and projected onto the television monitor screen (from Berns and Rounds, 1970; Berns, 1974).

The laboratory of Donald E Rounds at the Pasadena Foundation for Medical Research in California. lab was pioneering the use of the laser in a wide range of biomedical areas. One of these was the use of the microsecond-pulsed blue-green argon ion laser focused through a microscope to regions and organelles inside of live cells. Initially, combined with vital staining of specific cell organelles and subsequently without vital staining, individual chromosomes of live salamander mitotic cells were irradiated with the laser microbeam (Berns et al., 1969a, b). This system utilized a unique spinning mirror with a small hole, allowing a laser pulse to pass through the mirror (Figure 1; after Berns and Rounds, 1970; Berns, 1974). The capstone event of these early studies was demonstration that the less-than-a-micron diameter focused laser spot was capable of inactivation of the nucleolar (ribosomal) genes located in the secondary constriction (the nucleolar organizer region, *NOR*) on a single mitotic chromosome. The cells finished mitosis, and the daughter cells that received the chromosome with nucleolar gene ablation survived and had a reduced number of nucleoli commensurate with the number of ablated NOR’s (Berns et al., 1970). In a later study it was shown that the ribosomal gene site of ethidium bromide treated cells could be inactivated by two-photon absorption by the 1.06-micron wavelength of a 100 ps NdYag laser (Berns et al., 2000). These and previous studies on chromosome surgery were likely the first nanoscale use of multiphoton absorption in a biological system (Berns et al., 1981).

**FIGURE 2:**
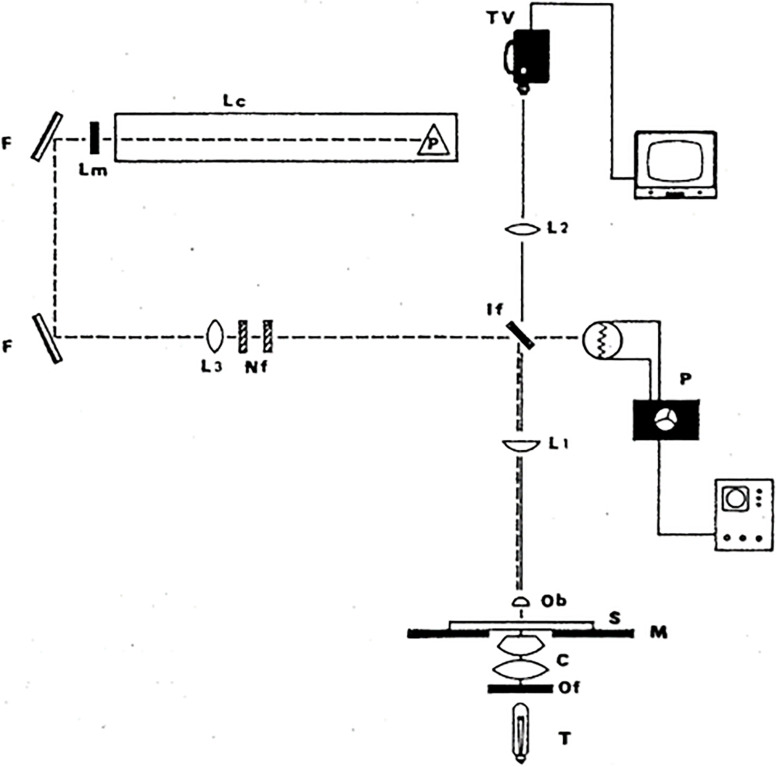
Simple and versatile argon ion laser microscope. Either of the two microsecond-pulsed 514 and 488 nm beams is passed into an upright Zeiss phase contrast microscope. It reflects 90 degrees off a dichroic interference filter (IF) that reflects the blue-green wavelengths into the microscope but transmits longer wavelengths to the video camera. The phase contrast objective (Ob) (100× or 63×; 1.4–1.3 na) focuses the beam to a diffraction-limited spot (0.4–0.5 μm) inside the target cell. Lc, laser cavity; Lm, laser output mirror; F, front surfaced reflecting mirrors; Nf, neutral density filter; If, dichroic interference filter; L1 and L2, lenses; P, photomultiplier dosimetry device; T, tungsten light source; c, condenser; Of, optical heat filter; M, microscope; S, microscope stage; TV, analog television camera (after Berns, 1971, 1974).

**FIGURE 3:**
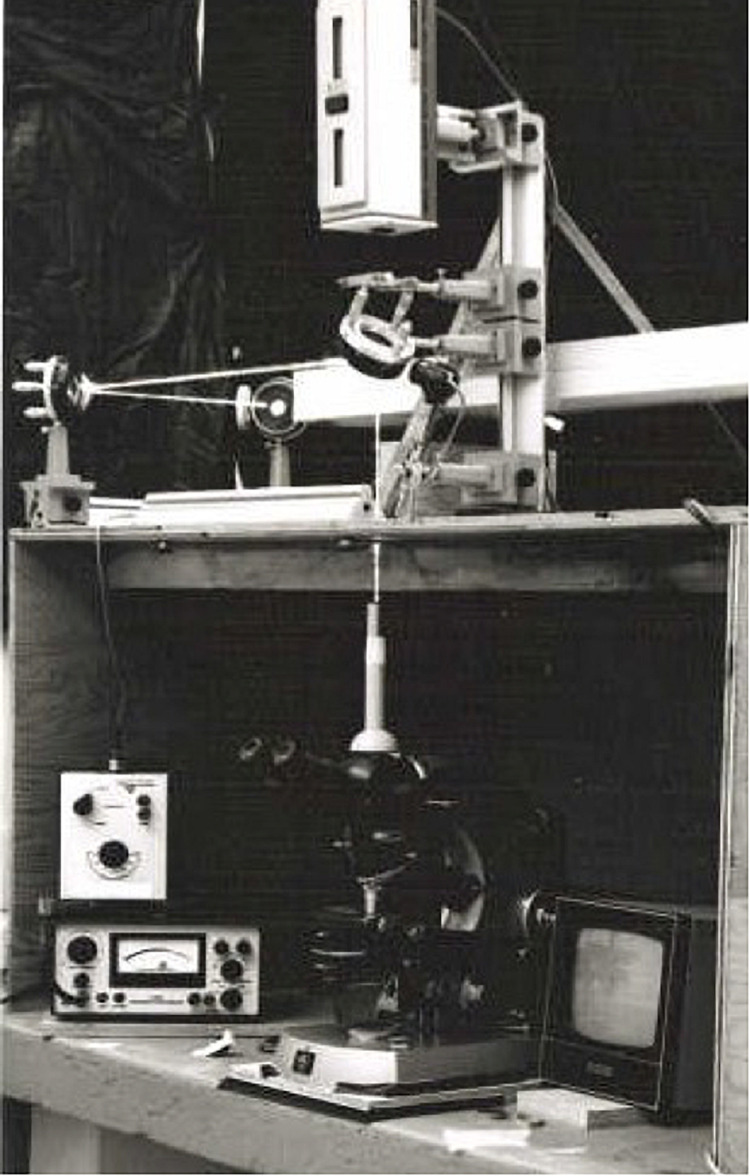
Argon ion laser microbeam depicted in Figure 2 (University of Michigan, Ann Arbor, 1971). This system was used for experiments from 1970 to 1979.

**FIGURE 4:**
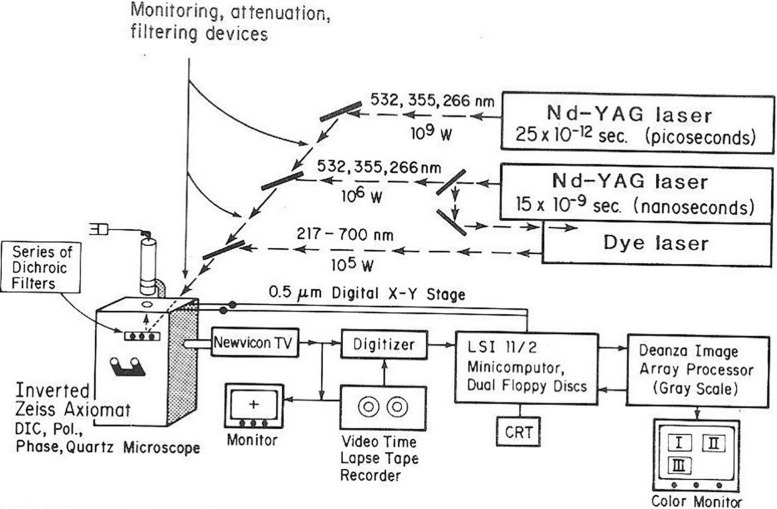
NIH LAMP P41 Biotechnology Resource laser microbeam system circa 1981. The lasers are a Quantel YAG 400 picosecond and nanosecond multi-harmonic device used to pump a tunable dye laser. The microscope is an inverted Zeiss Axiomat equipped with phase, bright field, polarization, and DIC optics. The digital imaging system uses a LSI-II driven Deanza IP 500 array processor interfaced with GYRR DA MKIII time-lapse videotape system. The LSI-II also controls the X-Y-Z microscope digital stage as well as image acquisition providing cell tracking capabilities. This system was replaced with the laser scissors-trapping system RoboLase system circa 2005 (see Figures 8, 9).

**FIGURE 5:**
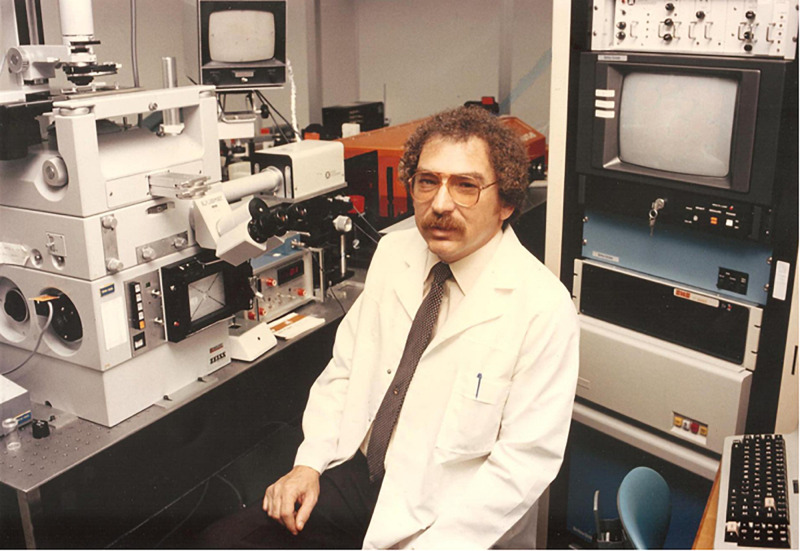
Photograph of system and author circa 1981. The computer behind the PI (principal investigator) is an LSI-11 driven Deanza image array processor. The microscope is a Zeiss Axiovert phase contrast, polarization system. The camera is digital imaging system which signal is sent to the computer system behind the PI. See Figure 4 for details about the system components. This system was used for experiments conducted between 1980 and 2000.

The argon laser microbeam was used first to produce half micron diameter lesions in individual chromosomes using the vital stain acridine orange (Berns et al., 1969a, b). Later, with higher energy density in the focused spot, the argon ion laser microbeam was able to alter chromosomes in live cells without any photosensitization (Berns, 1971; Berns et al., 1971a). In addition to eliminating side effects of the vital dye, this system replaced the spinning mirror with a dichroic filter that allowed the laser beam to reflect into the microscope, while at the same time projecting the image of the cell to a video camera above the dichroic (Berns, 1971, 1974). This simple and versatile laser microbeam is reproduced in Figures 2, 3, and was used for many studies until nanosecond and picosecond second, third, and fourth harmonic NdYag-dye lasers became available as in Figures 4, 5 (after Berns et al., 1981).

**FIGURE 6:**
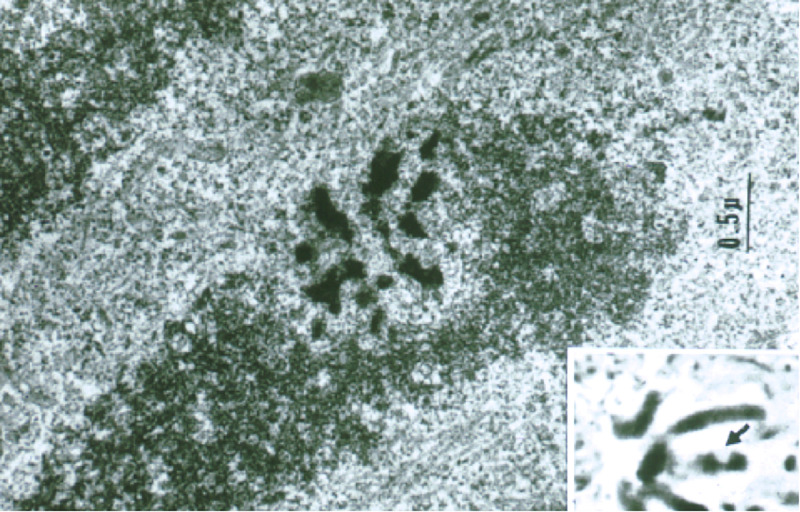
Transmission electron microscope (TEM) image of a laser-irradiated chromosome observed in a live mitotic cell by phase contrast (inset) and fixed, sectioned, and viewed by transmission electron microscopy. Note the phase-lightening of the laser lesion in the inset, and the matching region of the same chromosome in the TEM image. The 0.5–1 μm damage was confined to the laser irradiation spot (Rattner and Berns, 1974).

For the chromosome nano-ablation approach to be useful for long term studies, and specifically to functionally map gene regions, it was crucial to determine if the cells with a chromosome lesion could undergo subsequent cell divisions. In order to achieve this, we switched from primary lung cultures of salamanders, to the established cell lines of *Potorous tridactylus (PTK1 and PTK2)*, the Tasmanian rat kangaroo. These cells have only 10–12 large chromosomes and unlike most other vertebrate cells, the cells remained very flat during mitosis. This allowed the laser to be targeted to a specific site on an individual chromosome and was followed by electron microscope analysis to define the precise three dimensional localization of the lesion as in Figure 6 (Rattner and Berns, 1974). That these cells could undergo subsequent mitosis following production of a laser lesion on one chromosome arm was demonstrated using time-lapse 16 mm movies to follow a cell from time of irradiation in mitosis until a second mitosis 24–36 h later (Berns et al., 1971b). This experiment opened the door to studies following cells for hours and days after irradiation.

One of the first functional studies on PTK cells was to repeat the nucleolar organizer deletion study done in salamanders (Berns et al., 1972). In the PTK2 cells (a male cell line) there is one nucleolus per cell, and in PTK1 cells (a female cell line), there are two nucleoli per cell. The variation in number between male and female cells is due to the fact that the ribosomal (nucleolar) genes are on the X chromosome, so the female cells (PTK1) have two nucleoli per cell, and in the male cells (PTK2) there is one nucleolus (one NOR per cell). In one of the first studies, it was demonstrated that irradiation of one NOR in a female cell resulted in daughter cells with one nucleolus instead of two, and when the one NOR in male cells was irradiated, the cell compensated by making a large number of small micro-nucleoli. This was interpreted as indicating that removal of the major nucleolar organizer resulted in activation of secondary gene sites for nucleoli to facilitate cell survival. From an evolutionary perspective, this built-in redundancy made sense.

For the laser nano-ablation method to be generally useful, it was important to determine if a cell with a laser-induced chromosome deletion could be cloned into a viable population in which the deletion was maintained. This was achieved by isolating the irradiated cell and growing it into a viable clonal population (Basehoar and Berns, 1973). The question of whether or not a specific laser induced genetic deficiency could be maintained as a deficiency in a clonal population was determined by cloning cells with the selective reduction of one nucleolar gene site (NOR) (Berns et al., 1979, 1981). In these studies, cells with one laser-deleted nucleolar organizer region were cloned into viable populations. The reduction in nucleolar number was determined by cytological analysis of nucleolar number in clonal cells as well as analysis of karyotypes of Giemsa-banded chromosomes from clonal cells. These studies were unique in that, (1) they were one of the first microbeam studies employing the fourth 265 nm (UV) harmonic of a pulsed NdYag laser, and (2) the PTK cells irradiated were from a tetraploid cell line, thus facilitating the determination of the nucleolar and parallel chromosome deficiency of the nucleolar gene site. Surprisingly, it was determined that the nucleolar-deficient clonal cells had their normal complement of X-chromosomes, but the region of the secondary constriction (the site of the nucleolar genes) was deficient in one of the X-chromosomes (Berns et al., 1979). In a subsequent study, clonal cells with the laser-induced nucleolar deficiency were analyzed using DNA-RNA molecular hybridization. The results confirmed the earlier study, that the ribosomal DNA (the NOR) was missing from one of the X-chromosomes in the nucleolar-deficient clonal cells (Figure 7; Berns et al., 1979, 1981). The explanation of why cells with A and B a damaged chromosome that must have contained kilobases of damaged/deleted DNA can survive and replicate the chromosome that was laser-damaged was puzzling at that time. It took almost 40 years, and the blossoming of the field of DNA repair, to demonstrate that the laser-damaged X-chromosomes likely repaired the DNA to the extent that the DNA molecule maintained its structure as one linear molecule. Surprisingly, even though the irradiated X chromosome was replicated in the clonal cells, the deletion of NOR (rDNA) was maintained (Gomez-Godinez et al., 2020). This result, perhaps, explains why 21% of cells with a chromosome damaged in pro-metaphase and 37% of cells irradiated in anaphase were able to undergo subsequent cell divisions.

**FIGURE 7A:**
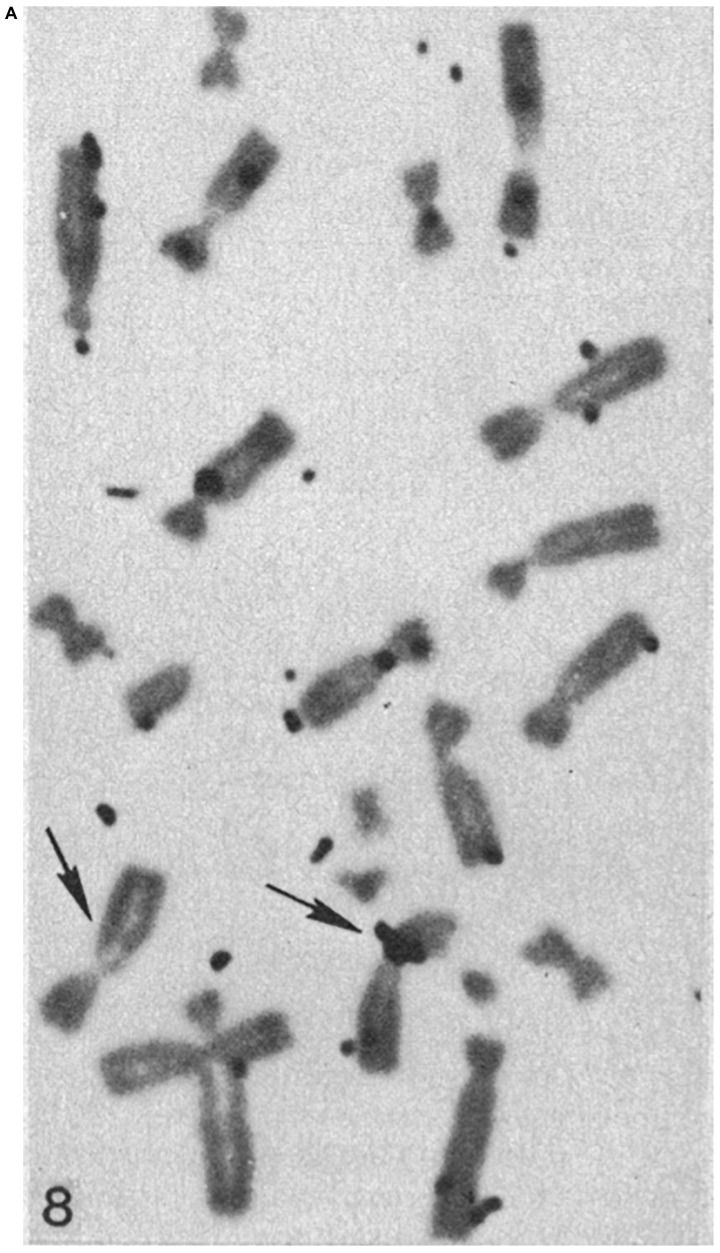


**FIGURE 7B:**
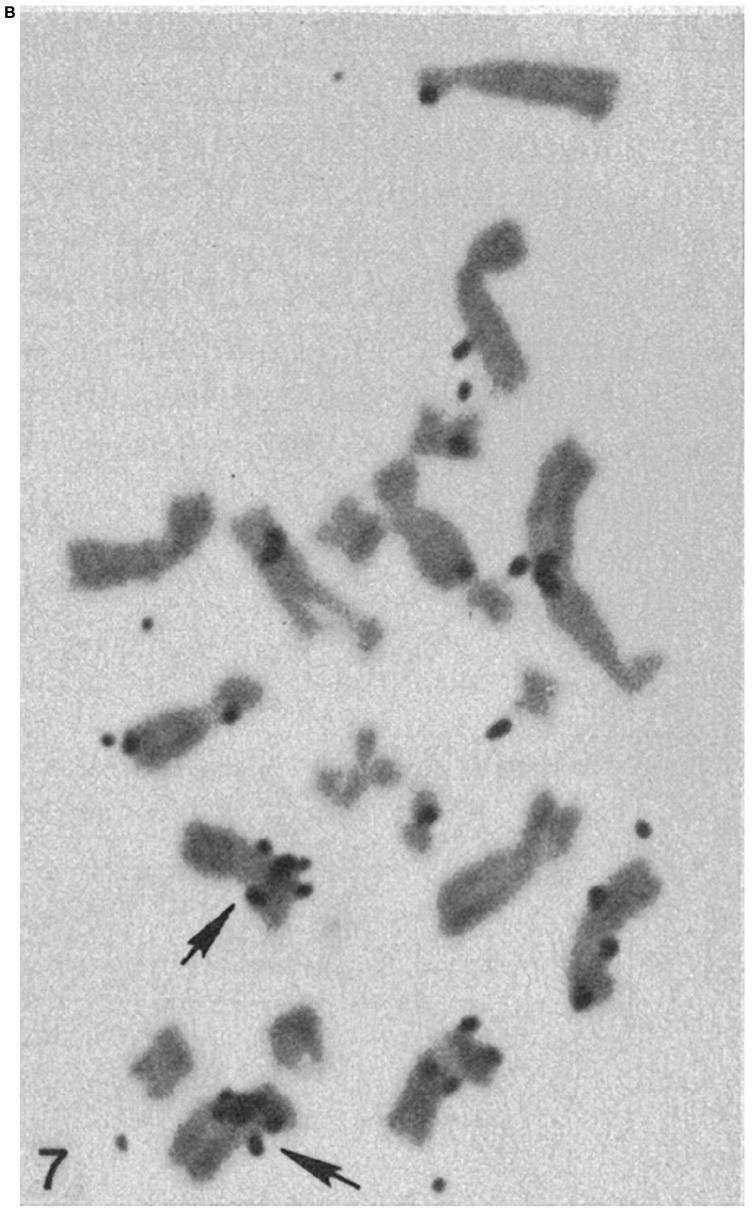
Chromosomes from hypotonic lysed tetraploid PTK2 cell clones subjected to H^3^-thymidine ribosomal RNA-DNA hybridization. The original parent cell of the cell in panel **(A)** had one NOR region ablated in mitotic pro-metaphase. The chromosome spread in panel **(B)** is from a control cell population that received no laser ablation. The cluster of autoradiography silver grains on a chromosome denotes the r-DNA NOR. The control tetraploid cell has two X-chromosomes, each with a cluster of grains at the cytologic location of the rDNA NOR, whereas the cell cloned from the laser-irradiated cell, has a cluster of silver grain only on one of the X-chromosomes, indicating that one NOR rDNA region has been deleted (arrows indicate the X-chromosomes; after Berns et al., 1979, 1981).

The 1981 *Science* review article (Berns et al., 1981), in addition to presenting a comprehensive review of the Berns’ -lab laser microbeam experiments over the previous 10 years, also describes the NIH LAMP biotechnology resource tunable nanosecond/picosecond Nd:YAG-dye laser microscope (Figures 4, 5). The system was coupled to a digital image array processor patterned after the groundbreaking work that the Jet Propulsion Laboratory, Pasadena, Ca. used for imaging the earth from space. This capability allowed for the first time, the use of real time digital processing to enhance images allowing for edge detection, contrast enhancement, and many other features afforded by digital manipulation of images (Walter and Berns, 1981). This work garnered two cover photos in *Science* (Berns et al., 1981; Altar et al., 1985). It was during this period that Karl Otto Greulich and colleagues in Jena, Germany developed their own system for laser microirradiation of cells and organelles. Over the years this group contributed numerous studies, including, many specifically targeting chromosomes (Monajembashi et al., 1986; Greulich, 1999; Berns and Greulich, 2007). These studies are summarized in several excellent reviews and books (Greulich, 1999, 2007, 2011, 2017; Berns and Greulich, 2007; Grigaravicius et al., 2009).

## Scissors II: Chromosome Movement in Mitosis/Meiosis

Concurrent with our genetic manipulation studies, we found that selective laser ablation of the kinetochore (centromere) region of a chromosome in either pro-metaphase or metaphase affected the movement of the irradiated chromosome (McNeill and Berns, 1981). This study demonstrated that destruction of the kinetochore on one side of a chromosome resulted in microtubule-mediated forces on the opposite side prematurely pulling the irradiated chromosome to the opposite pole. According to Magidson et al. in their 2008 review, “In a very influential paper, for example, McNeill and Berns (1981) showed, by selectively destroying just one of the two sister kinetochores on a prometaphase chromosome, that the velocity with which a kinetochore moves is independent of the mass associated with it. This study also implied that the mechanism that moves chromosomes during spindle assembly is the same that moves them poleward during anaphase.” In another study (Baker et al., 2010), selective irradiation of the telomere-tip of even a single chromosome after the onset mitotic anaphase movements resulted in all the chromosomes either delaying, stopping or reversing their anaphase movement, including reversal of the cytokinesis constriction. The net result was either a delay or cessation of the mitotic process. This result suggested a function for telomeres in regulation of cell division. Subsequent studies suggested that irradiation of telomeres caused initiation of DNA repair of the telomere region (Silva et al., 2014). It is likely that because of the importance of the telomere in the aging process and other cellular functions, immediate repair of the telomere-containing tips of chromosomes is essential. In many cells, there appeared to be a delay of the progression through mitosis until the DNA repair was complete. In other words, the biochemical machinery of the cell defers to repair of the damaged telomere before reactivating the molecular machinery for progression through mitosis.

In collaboration with Professor Arthur Forer, York University, Toronto, we conducted a multiyear series of studies on the forces involved in chromosome and spindle pole movement, in meiotic cells from several different invertebrates as well as in mitotic vertebrate PTK (*Potorous tridactylus*) cells. These studies suggested that in addition to the normal poleward forces exerted by kinetochore-attached microtubules, there are external non-spindle forces involved in chromosome movements (Forer et al., 2018). In particular, one source of force regulation on the chromosomes in meiosis of crane-fly spermatocytes appeared to be caused by tethers attached to the tips of separating chromosomes (Sheykhani et al., 2017). The results of these experiments indicated that the movements of partner anaphase chromosomes were coordinated by elastic tethers connecting the two chromosomes. In this study, when kinetochore microtubules were cut with the laser, the chromosome movements to the respective poles sped up, suggesting that the tethers function, in part, to regulate/control the speed of chromosome movement; the tethers act as an internal molecular governor. This study was followed by a subsequent study in which tethers connecting the tips of separating chromosomes were found in a wide variety of animal cells: *Mesostoma* (flatworm) spermatocytes, crane-fly spermatocytes (meiosis-I and -II), cricket spermatocytes (meiosis-I and -II), cellar spider spermatocytes (meiosis-I), black widow spider spermatocytes (meiosis-I), marsupial (PtK2) cells, and human U2OS (osteosarcoma) cancer cells. These results demonstrate that these structures likely play a role in coordinated chromosome movements in general, and must be considered in models of mitosis and meiosis (Forer et al., 2017). Furthermore, using optical tweezers, it was possible to calibrate the tethering force at 1.5 pN (Ono et al., 2017). Using optical tweezers to measure the force exerted on chromosomes is discussed below.

## Scissors III: DNA Repair

As mentioned previously, cells with damaged chromosomes were able to survive due to sufficient DNA-repair (DR). Laser induced DR is reviewed in this collection of papers by Kong et al. (in press). On a worldwide basis, a large number of labs that do these experiments use a variety of microscope laser systems with different wavelengths, pulse durations, and dosimetry. This variability, often makes it difficult to compare the results of one study with another, as reviewed in Kong et al. (2009). Notwithstanding this variability, it has become clear that laser microirradiation of the DNA/chromatin have contributed significantly to understanding the DNA damage recognition and repair processes. These studies have involved laser damage and repair in the interphase nucleus (Truong et al., 2013; Wang et al., 2013, 2014; Kim et al., 2015; Saquilabon Cruz et al., 2016; Kong et al., 2018; Murata et al., 2019), and on condensed chromosomes in mitosis (Gomez-Godinez et al., 2010; Silva et al., 2013).

## Tweezers

The first study using laser tweezers to move an organelle inside a live cell used a CW 1.06-micron wavelength NdYag laser according to the method invented by Ashkin and Dziedzic (1987). Our initial study demonstrated that isolated chromosomes from lysed *Potorous tridactylus* (PTK2) cells could be easily trapped and rotated using 10–100 mw of laser power in the focused laser spot. Using *F* = 2P/c, where P = laser power and c = speed of light, for the power used, this would be 6.7–133 × 10^–6^ dynes (Berns et al., 1989). This value converts to 67–1330 pN. In the same study, chromosomes inside live cells were exposed to the laser trap. Specifically, when the laser trap was applied to lagging chromosomes (chromosomes that were late to move toward the mitotic metaphase plate), rather than being pulled, as in the case of chromosomes in solution outside of the cell, the chromosome accelerated toward the metaphase plate, opposite from the direction of the pulling force. This result was interpreted as indicating that the mitotic spindle sensed the pulling force on the chromosome, and increased the rate of microtubule polymerization to pull the chromosome toward the rest of the chromosomes already aligned at the metaphase plate. This suggested that the mitotic spindle has the ability to modulate the forces applied to chromosomes in order for the cell to achieve equal distribution of chromosomes to its daughter cells.

**FIGURE 8:**
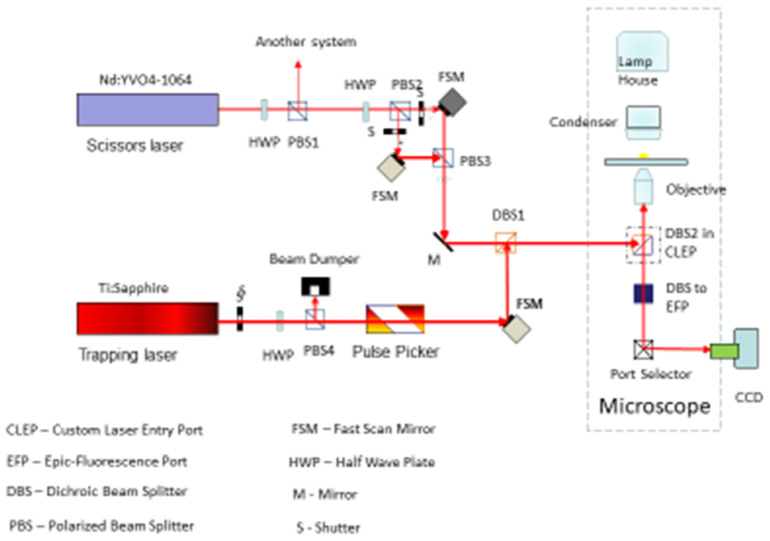
Combined laser scissors and trapping microscope. The trapping laser is a CW NdYag (1.06 μm) laser attenuted via a motor-driven rotatational half-wave plate. The beam is split by a beam-splitting polarizer into two trapping beams each controled by two fast scanning mirrors. This allows for two separately control trapping beams in the optical field. The ablation/cutting beam is a femtosecond Titanium sapphire laser tunable from 710 to 950 nm. The beam is attenuated by a half-wave plate and directed off a mirror and then off a fast scanning mirror. The beam is combined in the optical path with the trapping laser beam(s) thus allowing for separate control of either of the two trapping laser beams and the nano-ablation Titanium sapphire beam. Appropriate filter sets allow for reflection of one or more fluorescent wavelegth images of the cell into a cooled Hamamatsu ORCA CCD camera.

**FIGURE 9:**
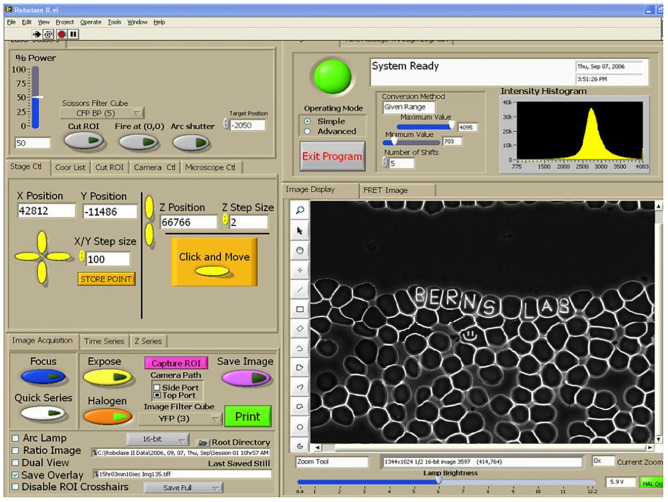
RoboLase control panel for system described in Figure 9. Air-dried human blood cells are visible in the image panel on the lower right. Name inscrcibed in individul red blood cells (8–10 micron diamter cells) illustrates the precison of the control of the focused laser lesion inside a single cell. Panel on left is used to control laser power delivered, x-y-z movement of the microscope stage, and controls to define the shape/geometry of the exposing laser beam. The control panel can be used in the lab containing the microscope system, or it can be used remotely from a lap-top/computer at another location, including in a foreign country, to conduct an experiment in real time via the internet (Botvinick and Berns, 2005).

Following the above study, a multiyear series of experiments was undertaken to explore and define the use of optical traps to study and manipulate chromosome movements in a variety of animal and plant cells. The following results/conclusions of these studies were made. (1) At the initiation of anaphase, a pair of chromatids could be held by the 1.06-micron NdYag laser optical trap and kept motionless throughout anaphase while the other pairs of chromatids separated and moved to opposite spindle poles. As a result, the trapped chromosome either was incorporated into one of the daughter cells, was lost in the cleavage furrow, or the two chromatids eventually separated and moved to their respective daughter cells (Liang et al., 1991). (2) Using a tunable CW Titanium Sapphire laser, the optical trap is stronger and more efficient because the wavelength transmission window of cells can be matched by the 700 nm laser wavelength for animal cells and 760 nm for fungal plant cells yielding stronger traps that have less secondary effects in both animal and plant cells (Berns et al., 1992). (3) The first combination of laser scissors and an optical trap (tweezer) involved using the laser scissors to cut chromosomes creating chromosome fragments. The optical trap was then used to hold the fragments while the cell progressed through mitosis, demonstrating the potential to use the two optical modalities to alter and manipulate chromosomes in live cells (Liang et al., 1993). (4) In another study on PTK cells, when the central spindle (the region between the separating chromosomes) was laser-cut, it was shown the central spindle was under tension generated by pulling forces in the asters (presumably MT-mediated) suggesting that the central spindle generates counterforces that limit the rate of pole separation (Aist et al., 1993). (5) A study was performed determining that the ability of cells to progress through mitosis was optimal when the chromosomes were exposed to trapping wavelengths from a tunable Titanium sapphire laser at 700 nm and 760–765 nm (Vorobjev et al., 1993). (6) Laser scissors and traps were used to demonstrate that the giant chromosomes of salamander lungs could be cut and moved on the mitotic spindle (Liang et al., 1994). (7) Laser microdissection of chromosome regions coupled with PCR analysis has been developed as a precise optical tool for genetic studies of Huntington’s disease (Hadano et al., 1991) and other gene sites on human chromosomes (He et al., 1997). In one recent study, a laser tweezers was used to move laser cut chromosome fragments inside the live cell to “weld” together new combinations of chromosomes (Huang et al., 2018). The above studies used a variety of lasers and optical systems and led to the development of a combined laser scissor and tweezers microscope that could be accessed through the internet using the LAMP Robolase control panel, making the technology available on a global scale, as in Figures 8, 9 (modified after Botvinick and Berns, 2005).

Of particular scientific importance is determination of the amount of force the spindle exerts on a chromosome during cell division (mitosis and meiosis). In subsequent studies, reviewing the results of the initial 1989 Berns et al. (1989) paper, two concerns in the force calculation were evident. First, the value of “2” in the equation for refractive index of the medium should really be 1.33 (the refractive index of water), and second, no value for *Q*, the trapping efficiency was used. This is significant since *Q* defines the amount of momentum in the focused laser spot that is translated to force on the object. Studies by Khatibzadeh et al. (2014) on isolated chromosomes in physiological saline have shown that under different viscosities, the values of *Q _Escape_* (the value of Q at which the chromosome drops out of the trap) vs. trapping powers (at < 20–60 mW), are 0.01–0.02. They also found little variation in *Q _Escape_* as a function of viscosity of the bathing solution.

Using *n* = 1.33 and *Q _Escape_* 0.01–0.02, subsequent experiments using a 50 mW 1.06-micron trap on chromosomes of dividing marsupial PTK2 cells, yields a trapping force of 2–17 pN to move a single chromosome in a mitotic cell (Liang et al., 1991). Subsequent trapping studies using appropriate *Q* coefficients for trapping efficiency gave the forces for stopping chromosome pole movements in invertebrate flatworm spermatocytes (*Mesostoma*) and crane-fly spermatocytes as 2–3 and 6–10 pN, respectively (Ferraro-Gideon et al., 2013). These forces are close to theoretical calculations of forces causing chromosome movements but orders of magnitude lower than the 700 pN measured previously in grasshopper spermatocytes in 1983 by Bruce Nicklas (Nicklas, 1983; Sheykhani et al., 2013). Additionally, as mentioned above, trapping experiments on isolated “*ex-vitro*” chromosomes from the mammalian Chinese hamster ovary (CHO) cells, in various viscosity solutions, confirmed theoretical force calculations of ≈ 0.1–12 pN to move a chromosome (Khatibzadeh et al., 2014). It is surprising that in light of the theoretical modeling, and the published laser trapping data, that the mitosis/meiosis establishment still accept that 700 pN is the amount of force exerted on a chromosome during cell division.

## Tweezers and Scissors

Since we first described the use of a laser scissors system in combination with a tweezers (Liang et al., 1993), very few studies using the two optical modalities combined have been conducted. However, one study used a pulsed violet laser scissors to cut chromosomes into small pieces and then used laser tweezers to move the cut piece to another chromosome where it was fused to create a chromosome with the added fragment (Huang et al., 2018). The authors suggested that this method “… provides a high quality alternative approach to directed genetic recombination, and can be used for chromosomal repair, removal of defects and artificial chromosome creation.” Though preliminary, it is hoped that this approach will, indeed, result in new ways to study chromosome behavior, as well as how the cell handles directed re-arrangement of chromosomes. The potential application to the study of chromosome-based congenital abnormalities is significant.

Another study combined laser scissors and tweezers to study the mechanical forces during mitosis (Ono et al., 2017). Optical tweezers and laser scissors were used to sever the tether between chromosomes, using the scissors to create chromosome fragments attached to the tether which move toward the opposite pole, and using the trap to stop the movements of the tethered fragments. When the telomere-containing region was severed from the rest of the chromosome body, the resultant fragment either traveled toward the proper pole (poleward), toward the sister pole (cross-polar), or movement ceased. Fragment travel toward the sister pole varied in distance but always ceased following a cut between telomeres, indicating the tether is responsible for transferring a cross-polar force to the fragment. Optical trapping of cross-polar traveling fragments placed an upper boundary on the tethering force of ∼1.5 pN. The discovery of tethers between the tips of chromosomes and the resultant cessation of chromosome fragment movement when the tethers were cut, provided convincing evidence that tethers need to be taken into account when describing and studying the forces and structures involved in chromosome movement during cell division.

## Discussion/Summary

**FIGURE 10:**
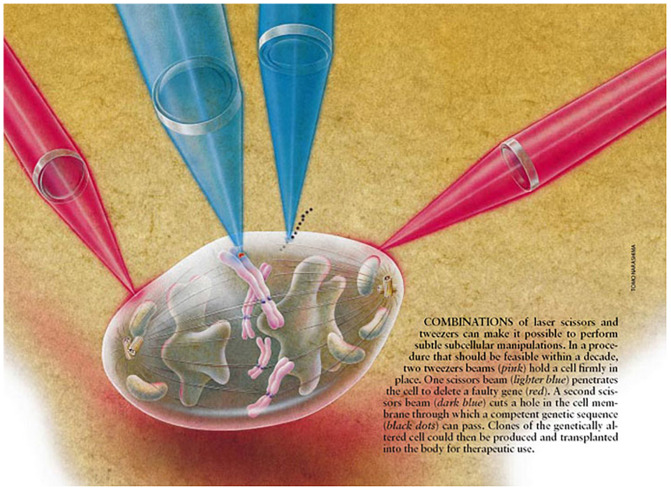
Laser scissors and tweezers conceptional schematic illustrating optical manipulation of a chromosome in a diviiding mitotic cell (after Berns, 1998).

Though laser scissors alone, optical trapping alone, and combined optical trapping and laser scissors have had impacts in many areas other than specifically on chromosome function and behavior as described in this review, chromosomes were one of the first target structures extensively studied with these modalities, and which continues today (see generic cartoon depiction, Figure 10, after Berns, 1998). Other articles and reviews contained in this compendium of *Frontiers’* papers on *Optical Trapping* (*Tweezers) and Nanoablation (Laser Scissors)* describe innovative studies contributing to this field. In this review I have focused on the research in my labs initially during my post doc period at the Pasadena Foundation for Medical Research under the guidance of Donald Rounds, at the University of Michigan as an assistant professor, the University of California Irvine in the Departments of Developmental and Cell Biology, Surgery, and Biomedical Engineering in the Beckman Laser Institute, and the University of California, San Diego in the Institute for Engineering in Research and the Department of Bioengineering. During these periods there have been many students and collaborators who have contributed to these studies, many of whom are listed in the papers cited in the reference section. In addition, visitors to my lab to use the NIH LAser Microbeam Program (LAMP) biotechnology resource center performed initial feasibility studies and then returned to their own labs to build laser microbeam systems of their own (see excellent review of the field of laser microbeams as well as of their application to study chromosome movements in cell division; Magidson et al., 2007).

**FIGURE 11:**
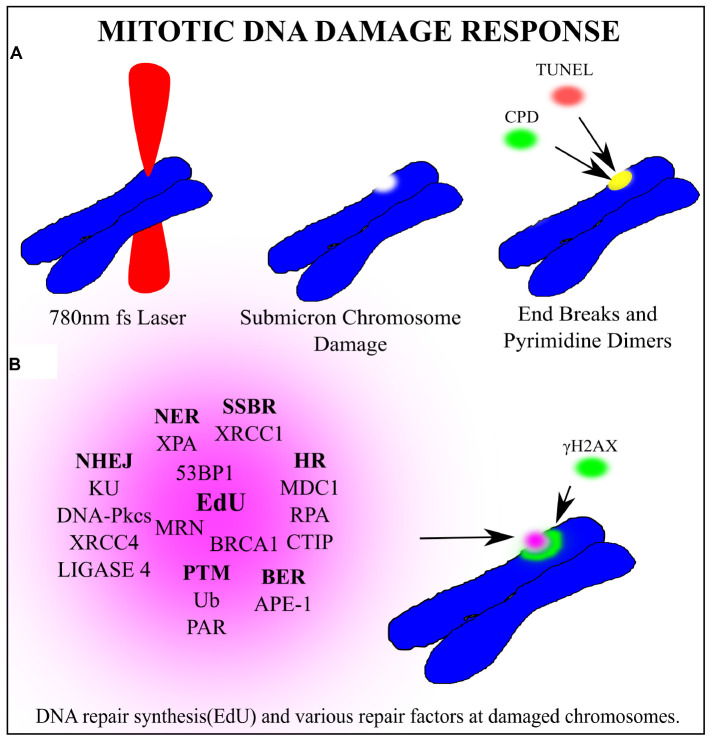
General schematic of a laser microbeam DNA repair experiment on a chromosome in a dividing cell. **(A)** A 780 nm femtosecond laser is focused to a sub-micron region on a mitotic chromosome. End breaks detected via TUNEL assay and cyclo-butane pyrimidine dimers were found at the laser damage site. **(B)** Several factors clustered to the damage site. In bold are the repair pathway abbreviations that each factor is most closely associated with. Non-Homologous End Joining (NHEJ), Single Strand Break Repair (SSBR), Base Excision Repair (BER), Homologous Recombination (HR) Post translational modifications (PTM), Nucleotide excision repair (NER). DNA synthesis occurs at the damaged chromosome region as detected via EdU incorporation. Phosphorylated Histone γH2AX on Serine 139 marks double strand breaks and extends from the laser damage spot. Based on the recruitment of the corresponding proteins, it is hypothesize that these repair pathways may be activated during mitosis.

Perhaps the one area where laser scissors (nanoablation) are routinely used by a significant number of labs around the world, is in the area of DNA repair. As reviewed in this article and elsewhere in this compendium of papers, a microscope-focused laser beam is used to produce either a single sub-micron damage spot on a single chromosome in mitosis and followed by studying the DNA repair process, or the focused laser is scanned through a micron-size linear track in an interphase nucleus (see cartoon depiction, Figure 11; Gomez-Godinez et al., 2020). As mentioned above, these studies have used a wide variety of lasers at different wavelength, energies, and focused spot size (Kong et al., 2009). This variability in studies is problematic because it is difficult to compare studies from different labs, thus standardization of parameters in the future is important.

Another area of concern is determination of the forces exerted on chromosomes during cell division. The laser tweezers studies to-date suggest the force to move a chromosome is in the 1–30 pN range. This is two orders of magnitude less that the classic mechanical measurements made in 1983 (Nicklas, 1983). Clearly this discrepancy will only be resolved when other investigators study this important question.

Finally, the combined use of scissors and tweezers offers unique non-invasive tools to study problems in cell biology and genetics. As described by Huang et al. (2018) as recently as 2018, these two photonic modalities can be combined to cut, move, and fuse chromosomes. The instrumentation used in that study compared to the complex system my colleagues and I developed (see Figure 8) is well within the capability and expertise of today’s bioengineer, cell biologist, and geneticist. Future studies using the combined technologies of the “optical toolbox” will continue to provide unique capabilities to study cells and their organelles.

## Author Contributions

MB was the sole author writing this article. Much of the work reported in this article represents the work of the author, his students, and collaborators over many years.

## Conflict of Interest

The authors declare that the research was conducted in the absence of any commercial or financial relationships that could be construed as a potential conflict of interest.
